# *In Vivo* Healing of Meniscal Lacerations Using Bone Marrow-Derived Mesenchymal Stem Cells and Fibrin Glue

**DOI:** 10.1155/2012/691605

**Published:** 2012-01-26

**Authors:** Dora Ferris, David Frisbie, John Kisiday, C. Wayne McIlwraith

**Affiliations:** Gail Holmes Orthopaedic Research Center, College of Veterinary Medicine and Biomedical Sciences, Colorado State University, 300 West Drake Road, Fort Collins, CO 80523, USA

## Abstract

Fibrin glue created from a patient's own blood can be used as a carrier to deliver cells to the specific site of an injury. An experimental model for optimizing various permutations of this delivery system *in vivo* was tested in this study. Harvested equine meniscal sections were reapposed with fibrin glue or fibrin glue and equine bone marrow-derived mesenchymal stem cells (BMSCs). These constructs were then implanted subcutaneously in nude mice. After harvesting of the constructs, BMSC containing constructs showed significantly increased vascularization, and histology showed subjectively decreased thickness of repair tissue and increased total bonding compared to fibrin alone constructs. This model allowed direct comparison of different meniscal treatment groups while using a small number of animals. This *in vivo* model could be valuable in the future to optimize fibrin and cellular treatments for meniscal lesions in the horse and potentially humans as well.

## 1. Introduction

The meniscus is a C-shaped fibrocartilage structure within the femorotibial joint [1]. It is integral in providing stability to the joint [2], lubrication during movement, and absorption and dissipation of shock [2, 3]. Meniscal injuries can result from a variety of causes, most notably chronic wear and tear, acute damage, and cruciate or collateral ligament laxity [4]. Over time, negative effects of meniscal injury include joint instability and articular cartilage degeneration. Medial meniscal injuries comprised 81% of the damaged menisci reported in humans, and sports activities were the major cause [5]. Severe meniscal tears in the horse have resulted in a decreased return to function despite surgical treatment [6, 7]. Meniscectomy and partial meniscectomy of severely damaged menisci are not without their long-term effects on the joint, resulting in osteoarthritis (OA) and joint disability [8]. Treatment or repair of the meniscus while avoiding meniscectomy may allow the patient to recover with decreased long-term dysfunction in the knee or stifle [9, 10].

 Efforts to create a synthetic replacement meniscus generally resulted in suboptimal performance of the implants with consequential degenerative joint changes in a matter of weeks to months in test subjects [11, 12] though some recent progress has been made [13]. Promising options have arisen using donor menisci as biologic scaffolding and incorporating these into the patient meniscal structure, or maintaining the patients' meniscus *in situ* and encouraging cellular repopulation and vascular in-growth through various methods [14–20]. Clinicians have begun to implant stem cells or platelet rich plasma into visible lesions via ultrasonography or arthroscopy. Fibrin glue derived from the patient's own blood can be used as a carrier to adhere the cells in the damaged region [17, 21]. A study in rabbits by Ishimura et al. [22] placed fibrin glue and whole bone marrow aspirate into defects in the avascular zone of the meniscus. This resulted in drastically more mature healing within 12 weeks than fibrin alone or empty defects. One version of this cellular/fibrin treatment has been pioneered by Scotti et al. [20] using chondrocytes implanted in fibrin glue to reappose cadaver meniscal sections. Their study showed increased bonding of the sections in the chondrocyte samples versus fibrin glue alone.

The aforementioned studies suggest the possibility that implanting viable cells in fibrin glue directly into a lesion may give rise to new advances in cell-based meniscal healing. Our laboratory has used intra-articular injection of autogenous bone marrow-derived mesenchymal stem cells (BMSCs) in the treatment of meniscal lesions in the horse for the last 4 years [23, 24] and has seen encouraging results for horses with severe meniscal lesions. Intra-articular injection of adult stem cells from bone marrow expansion showed significant regeneration of meniscal tissue in a caprine experimental model and a human clinical case [25, 26]. Intra-articular injection of BMSCs is not injury site specific, relying on inflammatory homing mechanisms [21, 27, 28] for the cells to migrate to the damaged tissues. The current project was developed to further examine the effects of more directed site-specific cellular treatment using fibrin as a carrier. Utilizing the murine model published by Scotti et al. [20] offers a variety of benefits that make it a suitable step between *in vitro *and larger scale *in vivo* work. The model provides the ability to experiment with meniscal constructs of a controlled size, type, and treatment in sufficient numbers with cohort controls to provide adequate statistical power. Such a model allows testing of various treatment permutations in a relatively short amount of time. These include dose (cell numbers), type or concentration of fibrin, degree of differentiation in multipotent cell lines, and addition of growth factors or other biologic substances in a controlled *in vivo* setting. The result will enable researchers to determine the optimal treatment strategies utilizing these modalities in larger animal models. The purpose of the current study was to determine if beneficial healing effects from implantation of BMSCs in fibrin could be observed compared to fibrin alone.

## 2. Materials and Methods

All procedures involving live animals and collection of tissue for this study were approved by the Colorado State University Animal Care and Use Committee.

### 2.1. Meniscal Tissue Collection

Eight medial menisci were collected from four horses euthanized for reasons unrelated to the stifle joint or other factors that would adversely affect the study. Menisci were harvested in an aseptic manner from cadaver limbs within 6 hours following euthanasia (with a range of 30 minutes to 6 hours), held in phosphate buffered saline (PBS) (Invitrogen Corporation (Headquarters) Carlsbad, CA, USA), snap frozen in liquid nitrogen, and then stored at −80°C for a minimum of 1 week (with a range from 1 week to 8 months). Samples were thawed in a 25°C water bath, trimmed to retain only the axial 2/3 of the meniscus, and sectioned further into 0.4 cm wide triangular wedges. Sections were placed into PBS and PSA (penicillin, streptomycin, and amphotericin B) (Invitrogen Corporation (Headquarters) Carlsbad, CA, USA) and passed through two additional freeze-thaw cycles [29], changing the media to fresh PBS (Invitrogen Corporation (Headquarters) Carlsbad, CA, USA) with each freeze-thaw cycle, to ensure that no viable cells were present.

### 2.2. Bone Marrow

Bone marrow was collected from the ilium of a horse euthanized at CSUVTH (Colorado State University Veterinary Teaching Hospital) for unrelated reasons within 30 minutes of euthanasia with an 8-gauge Jamshidi trocar using sterile technique. Approximately 20cc bone marrow was drawn into 2–35cc syringes with 3,000 units of heparin (APP Pharmaceuticals LLC, Schaumbeurg, IL, USA) anti-coagulant in each syringe. Bone marrow mesenchymal stem cell colonies were obtained by culturing the nucleated cell fraction according to the technique described by Kisiday et al. [30]. After reaching 60–70% confluence, each culture was passaged by lifting the BMSCs with trypsin (Invitrogen Corporation (Headquarters) Carlsbad, CA, USA), reseeding at 10,000 cells/cm^2^, and allowing the BMSCs to grow to 60–70% confluence. The BMSCs were cryopreserved in 95% autogenous serum/5% DMSO (Sigma Aldrich, St. Louis, MO, USA).

### 2.3. Fibrin

Four hundred milliliters of equine venous blood was collected in a sterile manner from the jugular vein of a donor into a blood collection set (Jorgensen Laboratories) with sodium citrate anti-coagulant and stored at 4°C for several hours. Whole blood was centrifuged at 1000 G for 10 minutes and the plasma removed and stored at −80°C until needed. Plasma was thawed at room temperature, and the fibrin extracted per the ethanol precipitation protocol described by Yoshida et al. [31].

### 2.4. Construct Preparation

Preprepared meniscal sections, BMSCs, and fibrin were thawed immediately prior to construct creation. Construct pairs for each mouse (Group 0 and Group 1) were created from sections of the same meniscus to control for variation. For Group 1 constructs, thawed cell suspension was pelleted, then resuspended with the minimal amount of fibrinogen free plasma (approximately 50 *μ*L) needed to return cells to suspension. The cell suspension was then mixed with 225 *μ*L fibrinogen precipitate and additional fibrinogen-free plasma (approximately 175 *μ*L) until a final cell concentration of 10 × 10^6^ cells per mL of fibrinogen/plasma/cell mixture was reached. Equal parts (0.02 mL) of the fibrinogen/plasma/cell mixture were mixed with an equal amount of bovine thrombin (MP Biomedicals, Santa Ana, CA, USA) (0.02 mL) and applied to the cut surfaces of Group 1 meniscal sections. A second meniscal section was placed on top of the fibrinogen/plasma/cell mixture and the fibrin allowed to set for 30 minutes.

Group 0 samples were created using 50 *μ*L of PBS to replace the BMSC/plasma suspension. This was then mixed with 225 *μ*L fibrinogen precipitate and 175 *μ*L of fibrinogen free plasma. Equal parts (0.02 mL) of the fibrinogen/PBS/plasma mixture were mixed with bovine thrombin (MP Biomedicals, Santa Ana, CA, USA) (0.02 mL) and applied to the cut surfaces of Group 0 meniscal sections. A second meniscal section was placed on top of the fibrin/PBS/thrombin mixture and the fibrin allowed to set for 30 minutes. To coat the exterior of all constructs, fibrinogen precipitate (225 *μ*L) was mixed with fibrinogen free plasma (225 *μ*L) and mixed with equal volumes of thrombin. This coating was applied to decrease subcutaneous tissue invasion *in vivo* [20]. Approximately 0.1 mL of fibrin and thrombin mixture was used to completely coat each construct. The exterior fibrin coating was allowed to set for 30 minutes before the completed constructs were transferred to a sterile dish for transport to the surgical suite. Constructs were assembled and completed in groups of 6 of each treatment group between 15 minutes and 45 minutes prior to implantation.

### 2.5. Surgical Procedure

Twelve 10-week-old male nude mice were anesthetized, maintained on isoflurane inhalant anesthesia, and administered 5 mg/kg carprofen (Pfizer Animal Health, Madison, NJ, USA) subcutaneously. Mice were placed in sternal recumbency and bilateral paralumbar areas were prepped with 2% Chlorhexidine scrub and sterile water. Surgical procedure was modeled after previously described protocols [20, 32]. A 1 cm horizontal cutaneous incision was made in each paralumbar area and a pocket bluntly dissected into the subcutaneous tissue. A meniscal construct was inserted into each pocket, one side randomly receiving a BMSC and fibrin-treated sample and a fibrin only control sample in the other [32]. The incisions were closed with wound closure clips (AUTOCLIP) (Becton, Dickson and Co. Franklin Lakes, NJ, USA). Ear notches were used to uniquely identify each mouse. Mice were recovered and were monitored twice daily for three days following surgery. Mice received 5 mg/kg of Carprofen subcutaneously for 3 days following surgery. After three days, they were monitored once daily for the remaining 4 weeks of the study for signs of postoperative complications and general well-being.

### 2.6. Harvesting of Constructs

At 4 weeks after surgery, mice were placed in a CO_2_ gas chamber and humanely euthanized with a gradually increasing concentration of CO_2_ (starting with room air) until the mice ceased to breathe. A lack of heart beat was confirmed before the mice were completely removed from the chamber. The constructs were removed, immediately fixed in 10% neutral buffered formalin (StatLab Medical ProductsMcKinney, TX, USA), and graded for presence of tissue adhesion, adherent vessels, and perceived strength of bond between the sections when bluntly probed. Twenty four meniscal samples in total were harvested from 12 animals, with a Group 1 and Group 0 harvested from each animal as modeled by Peretti et al. (2001) [32]. Grading scores and criteria are illustrated in the rubric in Table 1.

### 2.7. Scoring

A modified Rodeo et al. [33] scoring system was used. See Table 1 for the complete rubric. The outcome parameter “Adherent vessels” graded on a scale of 0–2 the extent of subcutaneous vascular adherence to each surface of the construct. The outcome parameter “Tissue adhesion” (scale 0–1) graded adherence of mouse subcutaneous tissue to the surface of the construct. The outcome parameter “Bond” scored the apparent adhesion between the two sections when gently probed along the junction of the two sections (0–2). A classification of “No bond” was determined if the two sections separated completely with gentle probing, “flexible bond” was determined as one that maintained apposition but the sections could be slightly shifted in relationship to each other, and a “firm” attachment was one where no movement was noted between the sections when probed.

### 2.8. Histology

Five micron sections were created from paraffin-embedded constructs in a plane perpendicular to the bonded edge. Two random tissue sections from the central area of each sample were selected and placed into two different staining groups. Tissues were applied to slides and stained with haematoxylin and eosin (H&E); (Anatech LTD; Battle Creek, MI, USA) and Safranin O-Fast Green (SOFG) ( Electron Microscopy Sciences, Hatfield, PA, USA) stain and examined microscopically.

 The outcome parameter “cellular ingrowth” graded the cellular infiltrate along the repair tissue interface to repopulate the acellular meniscal tissue (0–3). The outcome parameter “predominant cell type” assessed the population of cells present in the repair tissue and their relative frequency (1–3). The outcome parameter “fiber organization” graded the repair tissue as organized or disorganized (0–2 scale). The outcome parameter “fibrinous tissue between sections” graded the amount of fibrinous tissue noted between the meniscal sections at the time of analysis (0–1). This tissue was assumed to be remnants of the fibrin glue which was placed between the sections during construct creation. The outcome parameter “SOFG % positive staining” assessed the amount of the repair tissue that exhibited SOFG positive staining (0–3). The outcome parameter “thickness of repair tissue” assessed the thickness of the repair tissue that was present between the meniscal sections (0–2). The outcome parameter “cell repopulation” analyzed the presence of cells throughout the body of the meniscal section (0–2).

The outcome parameter “total percent bonded” was completed on a microscope at 20x–40x power and measured on digital capture images of the slide sections (Adobe Photoshop CS Extended Edition 10.0.1) Additional images for publication were obtained (Leica DFC 425 camera, LAS Core software, Buffalo Grove IL, USA). Slide images of the sections were measured for a total length of the repair area (area of interface between the cut sections). Images were then marked where evidences of bonding (bridging and incorporation of meniscal fibers into the repair tissue) were noted along the length of the repair. Bonded measurements were recorded and converted to a percentage of the total repair section. These total percentages were grouped into 4 groups and assigned a score of 0–3 based on Table 1.

### 2.9. Statistical Analysis

Fishers Exact Test and Chi-Square table analysis were performed on all data (SAS v.9.2., SAS Institute Incorporated, Cary, NC). Statistical significance was set at *P* ≤ 0.05 and a statistical trend was defined as *P* ≤ 0.1.

## 3. Results

There were no morbidities or mortalities in any of the study animals. At a 3 day post operative examination it was noted visually through the skin of two mice that two of the constructs had shifted so that sections were no longer in apposition on the cut surfaces (one was sitting edge to edge, one was sitting edge to cut surface). Both samples were in the cell-treated group (Group 1). Once cut in for histology, these samples had bonding evidenced between the sections and were graded along with the rest of the samples. For complete results of the scoring rubric for each sample, see Table 2.

### 3.1. Gross Observations

**Figure 1:**
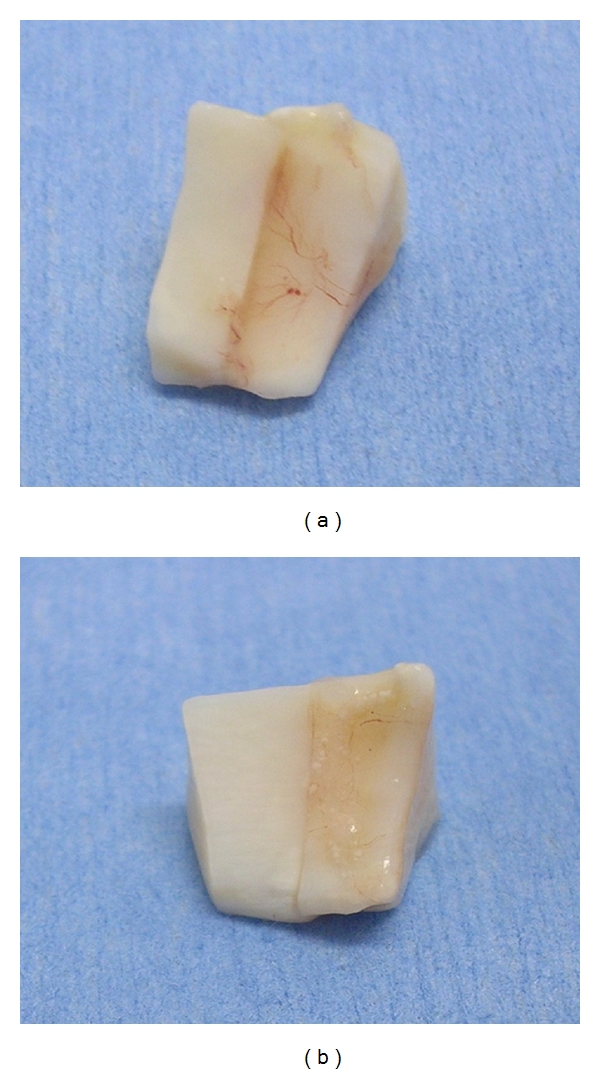
(a) Multiple vessels externally adhered to Group 1 meniscal construct. The vessels can be seen entering the cut area between the two sections. (b) Group 0 construct with minimal evidence of vessels on only one surface.

All constructs had fibrinous coating remaining, noted upon removal from the mouse. There was a statistically significant difference (*P* = 0.0094) in the presence of “Adherent vessels” to the construct surfaces. Group 0 had four (4/12, 33.3%) constructs with vessels present only on one side while Group 1 constructs all had vessels present on two sides (10/12, 83.3%) and multiple sides (2/12, 16.7%). Please see Table 2 for complete scoring results. See Figure 1(a) for an image of a Group 1 construct with multiple vessels adherent to the surface and Figure 1(b) for a Group 0 construct with minimal vessels present only on one surface. There was no statistical difference (*P* = 1.0) in external “tissue adhesion” between treatment groups. Group 1 constructs had no presence of tissue adhesion in 5/12 (41.7%) and did have presence of tissue adhesion in 7/12 constructs (58.3%). Group 0 also had 5/12 constructs with no evidence of external tissue adhesion and 7/12 constructs with presence of tissue adhesion. There was no significant statistical difference (*P* = 0.64) in the level of subjective of “Bond” between treatment groups. While some sections had a flexible bond, it was noted that all sections were adhered together and more resistant to separation with pressure applied at the cut edge than they had been prior to implantation, indicating the occurrence of bonding in all constructs. Please see Table 2 for a breakdown of the full results of these outcome parameters.

### 3.2. H&E-Stained Sections

**Figure 2:**
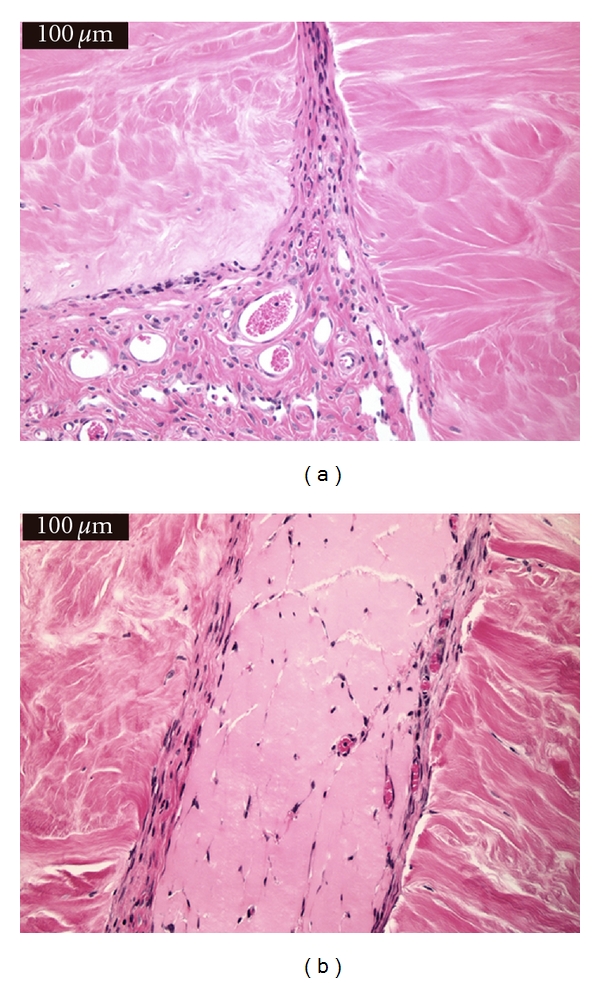
(a) shows thin repair tissue and vasculature ingrowth in Group 1 meniscal section. (b) shows a grouping of small vessels within a large amount of fibrinous tissue between two meniscal sections in Group 0 construct.

**Figure 3:**
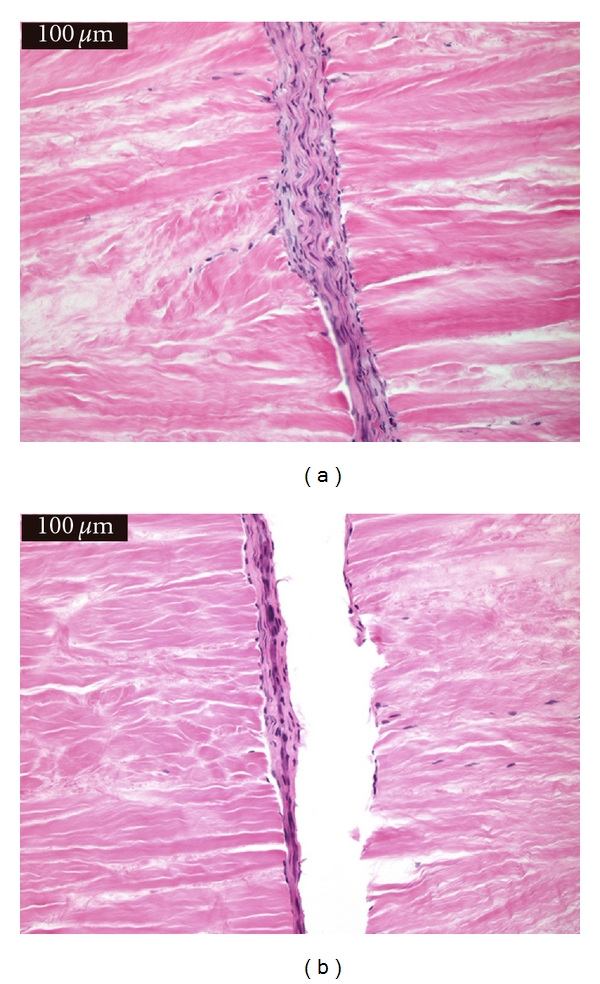
(a) A cell-treated (Group 1) meniscal section with cellular repopulation of the acellular meniscal tissue and evidence of bridging between the two meniscal sections, especially in the upper aspect of the image. (b) A non-cell-treated section (Group 0) with separation of the repair tissue from the meniscal fibers on the right-hand side (indicative of a less robust attachment). While this detachment occurred during processing, a lack of bonding can be seen on the left side of the repair tissue as well.

There was no statistical significance (*P* = 1.0) in “cellular ingrowth” from the repair tissue into the meniscal sections between treatment groups. There was no statistically significant difference (*P* = 0.822) in the “predominant cell type” present in the repair tissue between the treatment groups. Some sections had very organized repair tissue with parallel fibers; however, these parallel fibers were mostly oriented perpendicular to the meniscal fibers. While there subjectively appeared to be a relationship between treatment group and fiber organization, this was not statistically significant (*P* = 1.0). There was no statistical difference (*P* = 0.214) in the amount of fibrinous tissue remaining in the repair area between sections when comparing the treatment groups. Subjectively, larger amounts of fibrinous tissue were present in non-cell-treated sections than in cell-treated sections with 7/12 (58.3%) of the Group 0 constructs having a large amount of fibrinous tissue present compared to only 3/12 (25%) in the Group 1 constructs.  Figure 2(a) shows a histologic section from Group 1 illustrating the absent fibrinous tissue and showing vascular ingrowth into the repair tissue and progressing between the cut sections.  Figure 2(b) shows a histologic section from Group 0 showing thick fibrinous tissue present. There is still some vessel ingrowth present in the fibrinous tissue. There was no statistical difference in the “total bond distance” between treatment groups (*P* = 0.569). However, Group 1 (Figure 3(a)) subjectively showed better bonding overall with 11/12 (91.7%) sections exhibiting bonding characteristics evident in greater than 50% of the repair area compared to 9/12 (75.0%) of Group 0 (Figure 3(b)) constructs with bonding characteristics over 50% or more of the repair area.

### 3.3. SOFG-Stained Sections

**Figure 4:**
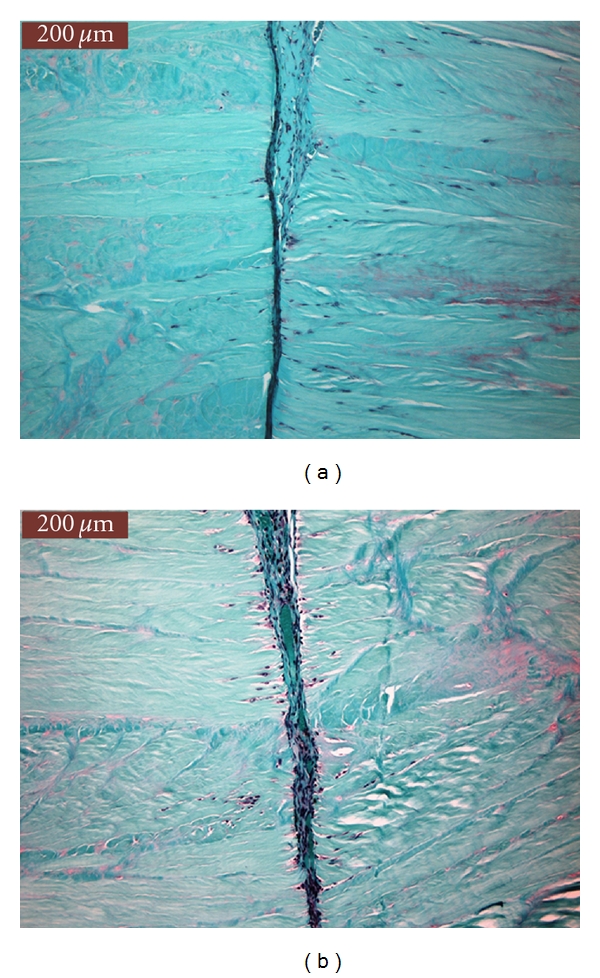
(a) and (b) show SOFG-stained sections with little positive SOFG-stain uptake. There was no significant differences between the SOFG staining between groups. Cellular repopulation along the cut edge is evident in both sections.

Figures 4(a) and 4(b) show two examples of SOFG-stained sections. There was no significant difference in “SOFG % positive staining” between groups (*P* = 0.428) with Group 0 exhibiting the same number of constructs with negative SOFG (i.e., green counter staining) (5/12, 41.7%) compared to Group 1 (5/12, 41.7%). While Group 0 had more constructs (4/12, 33.3%) that exhibited 50% or more positive SOFG staining compared to Group 1 (2/12, 16.6%), this difference was not significant. When examining “thickness of repair tissue” present and treatment group, there was no statistically significant difference between groups (*P* = 0.680). Based on subjective scoring, there appeared to be less repair tissue present between the sections in Group 1 (8/12 (66.7%) with thin repair tissue) compared to Group 0 (5/12 (41.7%) with thin repair tissue). Finally, there was no statistical difference noted when examining “cellular repopulation” in the body of the meniscal sections (away from the cut edge) between treatment groups (*P* = 0.520).

## 4. Discussion

This study allowed comparison of repair and bonding differences of acellular meniscal sections when treated with BMSCs and fibrin compared to fibrin alone in a controlled *in vivo* model. Overall, results showed significantly increased vascular adherence to BMSC and fibrin-treated sections (*P* = 0.0094). Subjectively the results showed improved characteristics of increased bonding and healing in constructs treated with stem cells and fibrin compared to constructs with fibrin only in the outcome parameters of “fibrinous tissue between sections”, “thickness of repair tissue”, and “total % bonded”. It is to be noted that the statistical results of this study are likely limited by subtle differences between the treatment groups and small sample size.

The degree of bonding seen in constructs treated with fibrin only in the current study appeared different when compared with previously published work. Specifically, Scotti et al. [20] saw no evidence of bonding between sections in their fibrin only group and did not report evidence of vascular infiltration in any of the experimental constructs in their study. In contrast, the current study showed multiple signs of bonding within the fibrin only treatment group as well as the BMSC-added group. There were vessels present between the sections in both treatment groups in the current study. It is unclear what resulted in this discrepancy between the results of the two studies. Potentially it could have been due to the difference in the fibrin sources. Scotti et al. [20] used a commercially prepared porcine fibrin product whereas the current study utilized equine fibrin drawn and prepared on-site, as would be commonly performed in a clinical setting. Another potential difference between the studies was the thickness of the fibrin coating. Images published by Scotti et al. [20] showed thicker layers of fibrin surrounding the constructs than was achieved in the current study. A thicker layer of fibrin would potentially limit vascular invasion and alter the diffusion and delivery of exogenous oxygen, cytokines, and cells to the construct.

In humans, while the outer third of the meniscus is well vascularized due to connections to the joint capsule, the axial portion is essentially avascular [34, 35]. Due to this difference, the outer portion of the meniscus is more likely to heal with a viable repair [34, 35]. The thinner avascular portion and meniscal ligament attachments are more likely to have a suboptimal repair and more likely to become reinjured on return to strenuous activity [14, 34]. Treatment of the constructs with BMSCs appeared to result in a greater and more consistent revascularization of the construct tissues. There were significantly (*P* = 0.0094) more vessels externally adhered to Group 1 constructs compared to Group 0 constructs in the current study. For an example, please see Figures 1(a) and 1(b). It is possible that vascular growth factors released by the BMSCs [21, 36, 37] resulted in a stimulus for vessel in-growth in the cell-treated sections, and these growth factors were not present in the previous study by Scotti et al. [20] which utilized chondrocytes in their cell constructs. It is also plausible that these growth factors may have also affected the non-BMSC (Group 0) sections implanted in the same mouse. Having both Group 0 and Group 1 constructs in the same mouse would allow growth factors to be systemically absorbed or to spread locally through the subcutaneous space. This may have accounted for the increased vascularity seen in the current study compared to Scotti et al. [20]. Increased vascular formation is often a goal of meniscal healing treatments in order to provide physiologic support for repair tissues in the meniscus. Thus the increased vascularity seen here could be a benefit in clinical use [38, 39].

Scotti at al. [20] reported more linear fibrous repair tissue and less fibrin remnant in the chondrocyte-treated constructs. These results are mirrored in the results of the current study with the BMSC-treated constructs: thinner repair tissue (66.6% of Group 1 compared to 41.75% of Group 0 constructs with thin repair tissue) and less fibrinous remnants (25% of Group 1 constructs had large amounts of fibrinous material between section compared to 58.3% in Group 0). One explanation for this result is that the cell-treated samples created a repair tissue with less extraneous scar tissue than those without cells.

Mature meniscus contains live fibrochondrocytes despite the avascular nature of the axial portion. If an injury occurs and viable cells are still present, these cells can be recruited for healing. However, studies have shown that fibrochondrocyte apoptosis is closely related to, and may even precede, meniscal damage [40]. Apoptosis that occurs in an injured area greatly reduces the fibrochondrocytes available for repair, and lack of vascularity limits the opportunity for cells to migrate in from other locations [41]. In a large retrospective study of humans with injured menisci, Englund et al. [10] found that degenerative meniscal damage was less likely to have a satisfactory return to function compared to traumatically injured menisci. Most of the constructs in both Group 0 (9/12) and Group 1 (9/12) in the current study had cellular ingrowth from the cut edge and repair tissue present. These cells could contribute to repopulation of the avascular meniscal section and ultimately increase healing. This repopulation of the acellular meniscal tissue would also be critical in healing as presence of fibrochondrocytes would be necessary for long-term viability of the meniscal implant. It was beyond the scope of this study to determine the origin of the repopulated cells.

The study presented here aided in further describing this model and resulted in significant results that may have clinical implications. Utilizing this model could allow further investigations to better define optimal conditions for fibrin and BMSC treatment in live animals. Unpublished studies *in vitro* in our laboratory have suggested that specific concentrations of fibrin may be optimal for migration of BMSCs; however, these more dilute concentrations may not offer as much support to maintain tissue apposition during the early healing phases. Using this model and cellular markers [38, 42] to track migration of cells at different time points may help to determine the most physiologically effective concentration of fibrin to utilize *in vivo*. Ideal dosage of cells per surface area treated may be investigated as well. Further studies with cell marking techniques will also provide information on the source of cells participating in the repair tissue between the sections and aid in determining what cells are repopulating the meniscal tissue. This may enable medical personnel to better target treatment to increase positive outcomes in difficult meniscal injuries.

## Figures and Tables

**Table 1:** Explanation of grading rubric used in the current study and the coding used for the results.

Category/score	0	1	2	3
Gross observations				
Adherent vessels	Vessels on 1 side only	Vessels on 2 sides	Multiple vessels on multiple sides	
Tissue adhesion	No Tissue Adhesion	Areas of external tissue adhered		
Bond	No bond	Flexible bond	Firm attachment	

H&E section observations				

Cellular in-growth	Acellular along cut edge	Incomplete cellular repopulation	50% in-growth	Complete repopulation along cut edge
Predominant cell type		Small round cells (fibrocytes)	Larger less dense cells (inflammatory origin)	50-50 dispersed
Fiber organization	Disorganized	50% parallel fibers	Greater than 70% organization	
Fibrinous tissue between sections	Large amounts of fibrous tissue present	No large fibrous tissue sections present		

SOFG section observations				

SOFG % positive staining	None	Less than 50%	More than 50%	Near 100%
Thickness of repair tissue		Thin	Medium	Thick
Cell repopulation	Rare	Moderate	Frequent	
Total bond distance	25%	25–50%	50–75%	75–100%

**Table 2:** Group 0 is fibrin only treatment; Group 1 is fibrin with BMSCs treatment. See Table 1 for grading score rubric.

Category/Score	Group	0	1	2	3	*P* value
Gross observations						
Adherent vessels	Group 0	4 (33.3%)	3 (25%)	5(41.7%)		0.0094
	Group 1	0 (0%)	10 (83.3%)	2 (16.7%)		
						
Tissue adhesion	Group 0	5 (41.7%)	7 (58.3%)			1.00
	Group 1	5 (41.7%)	7 (58.3%)			
						
Bond	Group 0		2 (16.7%)	10 (83.3%)		0.640
	Group 1		4 (33.3%)	8(66.7%)		

H&E section observations						

Cellular in-growth	Group 0	3(25.0%)	6 (50.0%)	3(25.0%)		1.000
	Group 1	3(25.0%)	5 (41.7%)	4 (33.3%)		
						
Predominant cell type	Group 0		8 (66.7%)	1(8.3%)	3(25.0%)	0.822
	Group 1		6(50.0%)	1(8.3%)	5(41.7%)	
						
Fiber organization	Group 0	3(25.0%)	7(58.3%)	2(16.7%)		1.000
	Group 1	2(16.7%)	7 (58.3%)	3(25.0%)		
						
Fibrinous tissue between sections	Group 0	7(58.3%)	5(41.7%)			0.214
	Group 1	3(25.0%)	9(75.0%)			
						
Total bond distance	Group 0	0	3(25.0%)	4(33.3%)	5(41.7%)	0.569
	Group 1	0	1(8.35)	7(58.3%)	4(33.3%)	

SOFG section observations						

SOFG % positive staining	Group 0	5(41.7%)	3(25.0%)	4(33.3%)	0	0.428
	Group 1	5(41.7%)	5(41.7%)	1(8.3%)	1(8.3%)	
						
Thickness of repair tissue	Group 0		5(41.7%)	6(50.0%)	1(8.3%)	0.680
	Group 1		8(66.7%)	3(25.0%)	1(8.3%)	
						
Cell repopulation	Group 0	5(41.7%)	6(50.0%)	1(8.3%)		0.520
	Group 1	7(58.3%)	3(25.0%)	2(16.7%)		
